# Harnessing subtractive genomics for drug target identification in *Streptococcus agalactiae* serotype v (atcc baa-611 / 2603 v/r) strain: An *in-silico* approach

**DOI:** 10.1371/journal.pone.0319368

**Published:** 2025-08-21

**Authors:** Ashiqur Rahman Khan Chowdhury, Farjana Yasmin Tithi, Nusrat Zahan Bhuiyan, Afsana Ferdousi Ishita, Md Mahmodul Hasan Sohel

**Affiliations:** Department of Life Sciences, School of Environment and Life Sciences, Independent University, Bangladesh; Albert Einstein College of Medicine, UNITED STATES OF AMERICA

## Abstract

Developing a therapeutic target for bacterial disease is challenging. *In silico* subtractive genomics methodology offer a promising alternative to traditional drug discovery methods. *Streptococcus agalactiae* infections depend on two crucial criteria: drug-resistance and the existence of virulence factors. It is essential to underline that *S. agalactiae* strains have emerged to be resistant to several drugs. Hence, there is a need for research on novel drugs and techniques that are potent, economical, productive, and dependable to combat *S. agalactiae* infections. In this study advanced computational techniques were exploited to examine potential druggable targets exclusive to this pathogen. Our study uncovered 200 non-homologous proteins in *S. agalactiae* serotype V (Strain ATCC BAA-611/ 2603 V/R) and identified 68 essential proteins indispensable for the bacterium’s survival. Therefore, these 68 proteins are potential targets for drug development. Subcellular localization analysis unveiled that the pathogen’s cytoplasmic membrane contained essential proteins among these vital non-homologous proteins. On the other hand, based on virulent protein predictions, six proteins were seen to be virulent. Among these, we prioritized two proteins (Sensor protein LytS and Galactosyl transferase CpsE which are exclusively found in *S. agalactiae*) as potential druggable targets and selected them for further structural investigation. The proteins chosen could serve as a foundation for the identification of a promising therapeutic compound that has the potential to neutralize these enzymatic proteins, thereby contributing to the reduction of risks linked to the drug-resistant *S. agalactiae*.

## Introduction

*S. agalactiae*, is a gram-positive, β-haemolytic coccus, commonly known as a group B streptococcus or GBS. This encapsulated facultative anaerobe is catalase-negative and features a group B antigen in its thick peptidoglycan cell wall, with a tendency to form chains [1,2]. It is commonly found as a normal part of the body’s flora in the urogenital and lower gastrointestinal tracts, and may also be present in the oropharynx [3–7]. Based on the composition of the capsular polysaccharide, a key virulence factor, this bacterium is classified into 10 serotypes (Ia, Ib, II-IX) [1,8–10].

In 1935, Rebecca Lancefield was the first to describe the colonization of the vagina by *S. agalactiae* and the first description of the bacterium as a human pathogen and its role in causing invasive illness was documented in 1964 [11,12]. It is mostly responsible for multiple infections including sepsis, meningitis, soft-tissue infection, urinary tract infection, pneumonia, and premature delivery [2,13–15]. Newborns of 24–48 hours old are more susceptible (approximately 33.3%) to this pathogen compared to those of older than 48 hours (only 8%) [16]. Among adults, this bacterium has the potential to induce peripartum chorioamnionitis and bacteremia in new mothers (occurring in 0.03% of cases) as well as endocarditis and osteomyelitis in other individuals [2,17]. Individuals who are immunocompromised, elderly, or have conditions such as diabetes mellitus, alcoholism, cancer, cirrhosis, a history of stroke, or HIV/AIDS are at increased risk of Group B streptococcal infection [2,17,18]. The pathogen continues to be a primary cause of newborn sepsis, resulting in around 90,000 fatalities in early infancy and at least 57,000 stillbirths worldwide [19].

Among the 10 identified serotypes, serotype V is recognized as one of the most clinically significant strains of *Streptococcus agalactiae* [20,21]. Its rising importance in clinical settings can be attributed to its evolving epidemiology, increasing virulence, and the development of antibiotic resistance. This serotype poses a particular threat in both neonatal and non-neonatal infections, as well as systemic conditions such as sepsis, meningitis, and pneumonia, where it is often linked to more severe clinical outcomes compared to other serotypes. Despite global similarities in *S. agalactiae* colonization rates, the prevalence of specific serotypes varies significantly across different regions [22]. Evidence suggests that serotype V has become dominant in several countries, including Algeria, France, Poland, Italy, Thailand, Brazil, Portugal, and the United States, although serotype III remains the most prevalent worldwide [23–30]. Studies also show that serotype III is more common in children, while serotype V predominates in adults and the elderly, often leading to both invasive and non-invasive infections [31]. The virulence of serotype V is largely attributed to its capsular polysaccharide (CPS), which plays a crucial role in its ability to evade the host’s immune system [32]. Serotype V strains, when compared to other serotypes such as I and III, have been shown to possess distinct virulence factors, including enhanced pili formation and biofilm production, which are key to their persistence and invasiveness [33–35].

A growing concern is antibiotic resistance in Gram-positive bacteria, including *Streptococcus* and strains of *Staphylococcus*. Treatment options are complicated by mechanisms such as β-lactamase production, efflux pumps, and target site modifications that reduce susceptibility to β-lactams, macrolides, and glycopeptides [36–38]. Additionally, in GBS, resistance mechanisms are driven by genetic mutations, horizontal gene transfer, and the acquisition of resistance genes from other bacterial species, which is often compounded by the widespread use of broad-spectrum antibiotics [34,35]. Studies have revealed that *Streptococcus agalactiae* strains exhibit enhanced resistance to a range of drugs, including erythromycin, clindamycin, tetracycline, fluoroquinolones, ampicillin, levofloxacin, cefotaxime, chloramphenicol, and vancomycin [20,23,26,27,29,39]. In some cases, resistance to penicillin, amoxicillin, ceftazidime, and piperacillin has also been observed in *S. agalactiae*, despite penicillin being the recommended first-line treatment for invasive GBS infections [40–43]. Given the escalating resistance of *S. agalactiae* to conventional antibiotics, identifying novel drug targets for *S. agalactiae* serotype V is crucial for the effective prevention and management of infections.

The exceptional advancements in computational biotechnology and bioinformatics have significantly impacted drug design, leading to reduced expense and duration for traditional laboratory trials by facilitating the discovery of therapeutic candidates, structure-based drug development, and the identification of host-specific targets through genomic data analysis. A key focus has been on the subtractive genomics technique, which aims to identify proteins with therapeutic potential exclusive to pathogenic genomes while excluding similar host proteins. This methodology has successfully identified novel species-specific therapeutic targets across various pathogenic strains including [44–63].

In this study, we aim to develop a potential therapeutic target to combat *S. agalactiae* serotype V through the application of sophisticated computational biotechnology and bioinformatics approaches, addressing the pressing global issue of drug resistance. Thus, in order to analyze the proteome of *S. agalactiae* serotype V in its entirety, the subtractive genomics technique was applied. Essential proteins for pathogen’s survival were prioritized using computational tools, followed by the removal of host homologous proteins to minimize therapeutic interference. The remaining pathogenic proteins were examined for subcellular localization and virulent capabilities, leading to the recognition of cytoplasmic membrane proteins and their virulence potential. Two promising proteins, Sensor protein LytS and Galactosyl transferase CpsE, were identified as potential therapeutic targets which are crucial for the virulence and pathogenicity of *S. agalactiae* serotype V. LytS regulates cell wall synthesis, stress response, and bacterial survival by maintaining cell wall integrity and mediating resistance to immune responses and antibiotics, whereas CpsE facilitates CPS biosynthesis, enhancing immune evasion by preventing phagocytosis and complement-mediated killing [32,64]. Together, these proteins contribute to the bacterium’s ability to cause severe infections, especially in neonates and pregnant women, by promoting tissue invasion and immune resistance. These enzymatic proteins underwent further protein network analysis and structural investigation, highlighting that they could potentially have the promise to serve as prospective selections for the development of vaccines or drugs that specifically target *S. agalactiae* serotype V.

## Materials and methods

**Fig 1:**
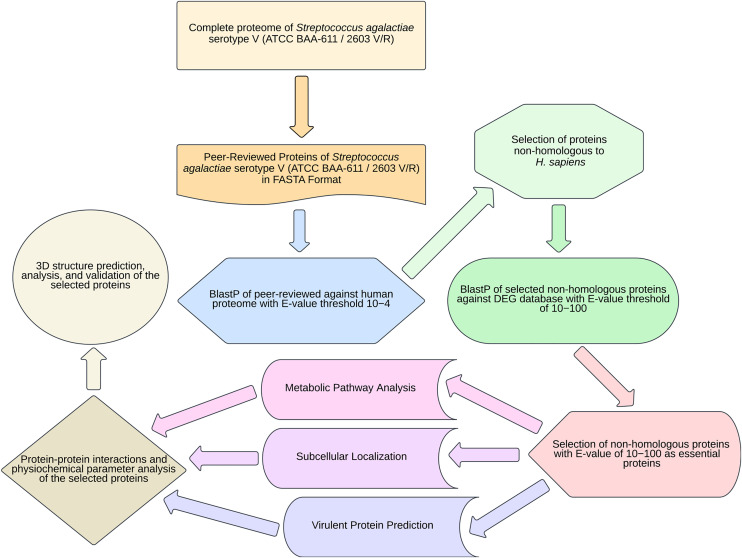
Flowchart for identifying potential drug targets in *S. agalactiae* serotype V. This shows the step-by-step process for identifying drug targets through the subtractive genomics approach.

The comprehensive protocol for discovering proteins that are essential and unique to *S. agalactiae* serotype V (Strain ATCC BAA-611/2603V/R) for potential drug target identification was achieved by recognizing non-human homolog proteins, their essentiality in the viability of the pathogen, their involvement in important pathogen-specific metabolic pathways, their sub-cellular localization, virulence, druggability, along with structural studies is outlined in Fig 1.

### Protein sequence retrieval

The whole proteome of *S. agalactiae* serotype V (Strain ATCC BAA-611/2603V/R) was retrieved from UniProt database [65–67] along with its peer-reviewed proteins in FASTA format.

### Finding non-homologous proteins

To distinguish proteins that are non-homologous to the host, a BLASTp search of the NCBI database (with e-value threshold of 10^−4^) was conducted against *Homo sapiens* on the selected peer-reviewed proteins of the pathogen [68]. The non-homologous protein sequences obtained were retrieved for further analysis and the rest having significant similarities with the host were excluded.

### Essential protein screening

Accordingly, the essential proteins among the non-homologous proteins were identified utilizing a BLASTp search (with a threshold e value of 10^-100^) against the Database of Essential Genes and protein sequences demonstrating notable similarity with the DEG database version 15.2 were categorized as being vital for the survival of the pathogen and were selected for subsequent analysis [69–72].

### Metabolic pathway exploration

The association of the chosen essential proteins in diverse metabolic pathways of the host and the pathogen was achieved by KEGG automated annotation server (KAAS) [73]. The three letter organism codes ‘sag’, ‘san’, and ‘sak’ for *S. agalactiae* and ‘hsa’ for *H. sapiens* were selected, and employing the BHH method in the KAAS server, the metabolic pathways were collected independently and compared.

### Subcellular localization prediction

Subcellular location of the chosen essential proteins was initially performed by the pSORTbv3.03 tool [74,75] and cross-verified by CELLOv2.5 [76,77]. Both tools provide accurate predictions for various subcellular locations, encompassing proteins found in the cytoplasm, cytoplasmic membrane, extracellular space, cell wall, and those with unknown origins [74–77].

### Virulent protein screening

Accordingly, virulent proteins from the shortlisted essential proteins were retrieved from the tool VirulentPred2.0 [78]. The tool utilizes up-to-date datasets and advanced machine learning techniques to forecast the virulence status of proteins based on their PSSM profile [78].

### Determination of physiochemical parameters

The ProtParam server of ExPaSy was used to determine the physicochemical properties of the selected proteins [79].

### Analysis of protein interaction networks

The potential interactions of the target proteins, with other proteins in *S. agalactiae* were predicted by the STRING database version 12.0 [80]. To prevent false-positive results, protein-protein interactions (PPI) studies were only considered if they had a high confidence score of 70% (0.700), choosing proteins with close interactions with at least three other proteins for further investigation.

### Druggability analysis

A BLASTp search with a threshold expectation value of 10^−5^ was performed on the shortlisted proteins against the DrugBank 6.0 database to evaluate their druggability [81]. Freely accessible, the DrugBank 6.0 database serves as a comprehensive online resource, providing extensive data on drugs and their respective targets [81].

### Prediction and assessment of three-dimensional structures

The three-dimensional structures of the target proteins were initially generated by the Swiss-Model online tool [82]. The tool uses homology-based approaches to construct 3D structures of proteins from amino acid sequences [82]. The 3D models of the selected proteins were constructed from template models that exhibited the most Global Mean Quality Estimate (GMQE) score, coverage, sequence similarity, range, and identity percentage [83–85] and was further validated by Swiss-Model structure validation (which included MolProbity scores, clash scores, and Ramachandran Plots) [86,87]. Moreover, we also utilized the SAVESv6.1 server (which included PROCHECK Ramachandran Plots and ERRAT scores) and the ProSAweb server (for Z-scores) to further verify and ensure the structural accuracy and quality of the constructed 3D structures [88–90].

### Active site prediction

We employed the DoGSiteScorer to effectively pinpoint potential active sites on the modelled protein structures where specific ligands could efficiently bind to it and modify its functions [91,92]. The server effectively identifies the present active site pockets by evaluating the physicochemical properties of the protein residues.

### Identification of ligands

Given that ligand information for the modelled proteins was unavailable, the ProBis server was employed to anticipate potential ligands and their interactions [93]. A theoretical methodology is employed by this server, which uses molecular simulations to anticipate the most suitable protein-ligand combinations. These results were subsequently visualized with PyMOL v3.1.

### Molecular docking studies

The AutoDock Vina in PyRx version 0.8 software was employed to dock the identified ligands with the modeled proteins serving as targets [94]. Using BIOVIA Discovery Studio version 4.5, the interactions between the ligands and the modeled proteins were then visualized.

## Results

Our study aimed to identify potential drug targets to combat the *S. agalactiae* serotype V strain, meeting the efficacy criteria of drug targets. The efficacy criteria include the targets must be non-homologous to humans, essential for the organism, and integral to the microbe’s main metabolic processes. Furthermore, membrane potential, virulence connections, and druggability analyses were also taken into account for strengthening the selection criteria of the target proteins. The subtractive genomic analysis process is briefly presented in Table 1.

**Table 1:** Brief presentation of the subtractive genomic analysis of *S. agalactiae* serotype V.

Sl No.	Subtractive Approaches	Bioinformatics Tools and Servers Utilized	Number of Proteins
1	The whole proteome of *S. agalactiae* serotype V (Strain ATCC BAA-611/ 2603 V/R)	UniProt	2105
2	Peer-Reviewed Proteins of *S. agalactiae* serotype V (Strain ATCC BAA-611/ 2603 V/R)	UniProt	390
3	Proteins nonhomologous to *H. sapiens*	BLASTp (E-value 10^−4^)	200
4	Essential proteins	DEG server (E-value ≤ 10^–100^)	68
5	Essential Proteins involved only in unique metabolic pathways	KAAS at KEGG	51
6	Proteins assigned KO (KEGG Orthology) but not in any pathway	KEGG Orthology	15
7	Essential membrane proteins	PSORTb	12
8	Essential membrane proteins	CELLO	8
9	Essential virulent proteins	VirulentPred2.0	6
10	Essential membrane and virulent proteins	PSORTb, CELLO, VirulentPred2.0	2

### Protein sequence retrieval

**Fig 2:**
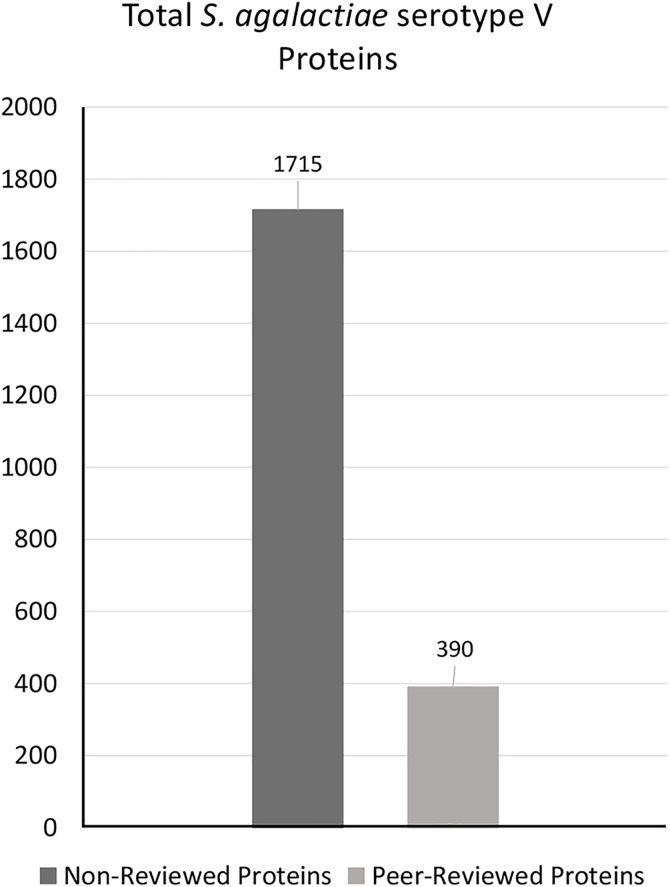
Total proteins of *S. agalactiae* serotype V. This shows the protein counts of non-reviewed and selected peer-reviewed sequences.

The total proteins available in the reference proteome of this strain in UniProt was 2105, among which 390 protein sequences were found to be Swiss-Prot (Peer-reviewed) proteins Fig 2. The study chose exclusively peer-reviewed sequences to avoid overrepresentation of specific proteins and computational difficulties, ensuring the accuracy and representativeness of the resulting data, thereby reducing redundancy within the proteome. For further analysis, all peer-reviewed proteins of the pathogen were collected in FASTA format, while excluding the non-reviewed proteins.

### Finding non-homologous proteins

Proteins that participate in various fundamental cellular systems have been identified as homologous, exhibiting analogous functions in both humans and bacteria throughout the course of evolution [95,96]. To identify non-homologous proteins, a BLASTp search of the NCBI database (with e-value threshold of 10^−4^) was conducted against *Homo sapiens* on 390 peer-reviewed proteins of the pathogen. Out of the 390 sequences, 200 were identified as non-homologous proteins, while the rest had resemblance to humans. We excluded the remaining proteins because cytotoxic reactions and adverse effects might arise from drug targets that are similar to the host genome and the aim of targeting such homologous proteins might result in detrimental consequences [97,98]. While homologous protein sequences were excluded, these 200 non-homologous proteins were chosen for further analysis.

### Finding essential proteins

To identify essential proteins of the pathogen, a BLASTp search against the database of DEG (with an e-value cut off at 10^-100^) was performed. Furthermore, to prevent false positive results, a manual cross-checking of the biological function of each query target protein was performed against its corresponding outcome, revealing that their functions are consistent. A total of 68 proteins (S1 Table) out of 200 were identified as crucial to the bacterium’s survival and were chosen for subsequent steps.

### Metabolic pathway exploration

The KAAS server revealed that 51 out of 68 essential proteins are associated with 41 distinct pathogen-specific metabolic pathways, with no proteins involved in host pathways. Moreover, no common pathways between the host and the pathogen were observed, leading to the conclusion that the selected non-homologous proteins showed no involvement in the host pathways. The absence of shared pathways between the pathogen and host suggests distinct metabolic systems, making pathogen-specific pathways ideal targets for drug development. Focusing on these unique pathways can effectively inhibit the pathogen without harming the host, reducing toxicity and improving the safety and efficacy of treatments. Furthermore, to manage and prevent false positives, stringent filtering criteria were applied such as essential proteins (e-value cutoff of 10 ⁻ ¹⁰⁰) and non-homologous (BLASTp e-value < 10 ⁻ ⁴). Functional annotation was performed using KO identifiers, ensuring accurate pathway mapping and manual comparison of pathogen vs. host pathways further evaluated and validated the metabolic pathways. Moreover, all 68 essential proteins had KEGG Orthology (KO) identifiers (S2 Table) by the KAAS server at KEGG, except for two proteins (namely, Bis(5’-nucleosyl)-tetraphosphatase (symmetrical) and galactosyl transferase CpsE). However, it is important to note that the absence of KO annotation for these two proteins does not imply false positives as Galactosyl transferase CpsE is a well-documented enzyme involved in capsular polysaccharide biosynthesis, a key factor in bacterial virulence and immune evasion [32], whereas Bis(5’-nucleosyl)-tetraphosphatase (symmetrical) plays a critical role in nucleotide metabolism and signaling by hydrolyzing dinucleoside polyphosphates, molecules known to regulate bacterial stress responses [99]. Limited KO database coverage for these proteins likely indicates annotation gaps rather than errors in target identification, and their essential, pathogen-specific roles validate their inclusion as targets. The unique metabolic pathways are listed in Table 2.

**Table 2:** Metabolic pathways unique to *S. agalactiae* serotype V.

Sl. No	Unique Pathways	Total Proteins	Pathway ID	Proteins Involved
1	Pentose phosphate pathway	1	00030	DEOB_STRA5
2	Amino sugar and nucleotide sugar metabolism	2	00520	MURA_STRA5, MURB_STRA5
3	Pyruvate metabolism	2	00620	ACKA_STRA5, CAPP_STRA5
4	Propanoate metabolism	1	00640	ACKA_STRA5
5	Oxidative phosphorylation	2	00190	ATPD_STRA5, PPAC_STRA5
6	Photosynthesis	1	00195	ATPD_STRA5
7	Carbon fixation by Calvin cycle	1	00710	CAPP_STRA5
8	Other carbon fixation pathways	2	00720	CAPP_STRA5, ACKA_STRA5
9	Methane metabolism	2	00680	CAPP_STRA5, ACKA_STRA5
10	Fatty acid biosynthesis	2	00061	FABH_STRA5, FABZ_STRA5
11	Glycerolipid metabolism	2	00561	PLSX_STRA5, PLSY_STRA5
12	Glycerophospholipid metabolism	1	00564	PLSY_STRA5
13	Purine metabolism	1	00230	DEOB_STRA5
14	Pyrimidine metabolism	3	00240	PYRH_STRA5, KCY_STRA5, KTHY_STRA5
15	Glycine, serine and threonine metabolism	1	00260	KHSE_STRA5
16	Cysteine and methionine metabolism	1	00270	KHSE_STRA5
17	Phenylalanine, tyrosine and tryptophan biosynthesis	4	00400	AROB_STRA5, AROD_STRA5, AROA_STRA5, AROC_STRA5
18	Taurine and hypotaurine metabolism	1	00430	ACKA_STRA5
19	D-Amino acid metabolism	4	00470	ALR_STRA5, DDL_STRA5, MURI_STRA5, MURD_STRA5
20	Peptidoglycan biosynthesis	7	00550	MURA_STRA5, MURB_STRA5, MURC_STRA5, MURD_STRA5, DDL_STRA5, UPPP_STRA5, MURG_STRA5
21	Teichoic acid biosynthesis	1	00552	UPPP_STRA5
22	Thiamine metabolism	1	00730	RSGA_STRA5
23	Nicotinate and nicotinamide metabolism	1	00760	NADE_STRA5
24	Pantothenate and CoA biosynthesis	1	00770	COAD_STRA5
25	Biotin metabolism	1	00780	FABZ_STRA5
26	RNA polymerase	1	03020	RPOA_STRA5
27	Ribosome	3	03010	RS3_STRA5, RS4_STRA5, RL10_STRA5
28	Aminoacyl-tRNA biosynthesis	2	00970	SYGA_STRA5, SYGB_STRA5
29	Protein export	1	03060	SECA_STRA5
30	RNA degradation	1	03018	RNY_STRA5
31	DNA replication	1	03030	RNH3_STRA5
32	Nucleotide excision repair	1	03420	UVRC_STRA5
33	Mismatch repair	1	03430	EX7L_STRA5
34	Homologous recombination	5	03440	RECF_STRA5, RECO_STRA5, RECR_STRA5, RECA_STRA5, RUVA_STRA5
35	ABC transporters	1	02010	PSTS1_STRA5
36	Bacterial secretion system	1	03070	SECA_STRA5
37	Two-component system	3	02020	PSTS1_STRA5, DNAA_STRA5, LYTS_STRA5
38	Cell cycle – Caulobacter	2	04112	DNAA_STRA5, MURG_STRA5
39	Quorum sensing	1	02024	SECA_STRA5
40	Tuberculosis	1	05152	PSTS1_STRA5
41	Vancomycin resistance	3	01502	DDL_STRA5, ALR_STRA5, MURG_STRA5

### Subcellular localization prediction

**Fig 3:**
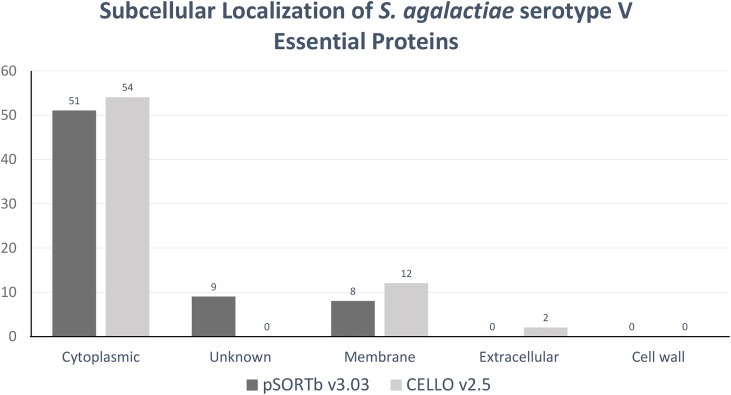
Essential, non-homologous proteins and their subcellular localization by pSORTbv3.03 and CELLOv2.5. This shows the protein counts of sequences predicted by subcellular localization prediction.

The pSORTbv3.0.3 tool predicted eight cytoplasmic membrane proteins, 51 cytoplasmic proteins, and nine proteins with uncertain or unknown subcellular localization from the shortlisted essential proteins. Subsequently, CELLOv2.5 predicted 12 membrane proteins, 54 proteins as cytoplasmic, and two extracellular proteins. However, both CELLOv2.5 as well as pSORTbv3.03 did not exhibit any proteins belonging in the cell wall region (S3 Table). The predicted results of the subcellular localization of essential proteins of *S. agalactiae* are presented in Fig 3. Out of the 8 and 12 cytoplasmic membrane proteins predicted by pSORTbv3.03 and CELLOv2.5, respectively, five proteins were predicted by both tools (Table 3).

**Table 3:** List of cytoplasmic membrane proteins identified by both pSORTbv3.03 and CELLOv2.5.

Sl No.	Gene name	Proteins	UniProt ID	pSORTb v3.03	CELLO v2.5
1	plsY	Glycerol-3-phosphate acyltransferase	P67167	Membrane	Membrane
2	ftsK	DNA translocase FtsK	Q8CX05	Membrane	Membrane
3	uppP	Undecaprenyl-diphosphatase	Q8E260	Membrane	Membrane
4	lytS	Sensor protein LytS/ Sensor histidine kinase LytS	Q8E218	Membrane	Membrane
5	cpsE	Galactosyl transferase CpsE	Q9AFI0	Membrane	Membrane

### Prediction of virulent proteins

Virulent factors in pathogens facilitate bacterial adhesion, colonization, invasion, and disease pathogenesis [100,101]. The identification of virulent proteins, among 68 essential non homologous proteins, using VirulentPred2.0 revealed six proteins that are listed in Table 4. Inhibition of these proteins could significantly impact the pathogen’s functionality within the host organism. Among the six predicted proteins, LytS and CpsE were found to be both virulent and present in cytoplasmic membrane which implies that these two proteins are potential drug targets Table 4 (Bold and Italic).

**Table 4:** Essential, non-homologous proteins predicted to be virulent by VirulentPred2.0.

Sl No.	Gene name	Proteins	UniProt ID	KO ID	VirulentPred2.0
1	hslO	33 kDa chaperonin	P64402	K04083	Virulent
2	ruvA	Holliday junction branch migration complex subunit RuvA	Q8DWW6	K03550	Virulent
3	pstS	Phosphate-binding protein PstS	Q8DZV4	K02040	Virulent
4	***lytS***	***Sensor protein LytS***	Q8E218	K07704	Virulent
5	recO	DNA repair protein RecO	Q8E2G8	K03584	Virulent
6	***cpsE***	***Galactosyl transferase CpsE***	Q9AFI0	–	Virulent

### Physiochemical property analysis

The physiochemical properties of the selected two proteins, namely Sensor protein LytS and Galactosyl transferase CpsE, were analyzed by ProtParam tool. The analysis showed that both proteins are slightly hydrophobic, basic, stable, and highly thermostable in nature. It is important to note that Galactosyl transferase CpsE is more basic in nature than Sensor Protein LytS. The detailed results of physiochemical property analysis of two proteins are presented in Table 5.

**Table 5:** ProtParam analysis of Sensor protein LytS and Galactosyl transferase CpsE.

Physicochemical Properties	Sensor protein LytS	Galactosyl transferase CpsE
Amino acids	581	449
Molecular weight	64548.62	52364.13
Theoretical pI	7.71	9.34
Instability Index	35.59	33.46
Aliphatic Index	107.01	103.94
Grand average of hydropathicity (GRAVY)	0.075	0.059
Estimated half-life in Mammalian reticulocytes, in-vitro	30 hours	30 hours
Estimated half-life in Yeast in-vivo	*>*20 hours	*>*20 hours
Estimated half-life in *Escherichia coli*, in-vivo	*>*10 hours	*>*10 hours

### Analysis of protein interaction networks

**Fig 4:**
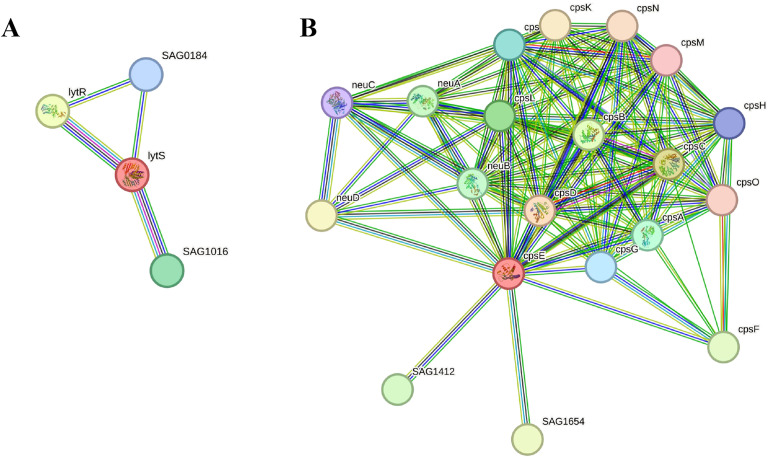
Prediction of protein-protein interactions of the two proteins. The chosen proteins are represented in red nodes. Each node represents all the proteins produced by a single, protein-coding gene locus. Empty nodes represent proteins of unknown 3D structure and filled nodes indicate that a 3D structure is known or predicted. Edges represent protein-protein associations. **(A)** Sensor protein LytS protein. **(B)** Galactosyl transferase CpsE protein.

The significant association of these two chosen proteins with other proteins in the pathogen was identified using the STRING database, and the amino acid sequences of Sensor protein LytS and Galactosyl transferase CpsE was uploaded to the server. The Sensor protein LytS developed 4 PPI networks (Fig 4A) depicted as lytS in red node. LytS had 4 edges, 3 anticipated edges, 4 nodes, with a 2.0 average node degree. Protein-Protein Interaction enrichment p-value was 0.331, with 0.833 average local clustering coefficient. Moreover, it interacted with two neighbouring two-component system response regulator proteins (lytR and SAG1016) and one neighbouring conserved hypothetical protein (SAG0184). On the other hand, Galactosyl transferase CpsE had 20 PPI networks (Fig 4B) represented as cpsE in red node. There were around 20 nodes, 125 edges, with an average node degree of 12.5, a clustering coefficient of 0.888, 21 predicted edges, and a PPI enrichment p-value of more than 1.0e-16. Furthermore, it interacted with numerous neighbouring proteins mostly involved in the assembly, regulation and biosynthesis of the GBS capsular polysaccharide (cpsD, cpsC, cpsB, cpsL, cpsA, cpsJ, cpsG, cpsH, cpsM, cpsO, cpsN, cpsK, cpsF) along with different other neighbouring important enzymes (neuC, neuD, neuA, neuB, etc).

### Druggability analysis

Binding of a small molecule, such as a drug or vaccine, to a protein can induce a functional change, thereby converting the protein into a “druggable target.” This transformation typically alters the protein’s activity, resulting in therapeutic effects that enhance the health or condition of the host organism [102]. Therefore, a BLASTp analysis was performed on the two selected proteins using an e-value threshold of 10^−5^, against the DrugBank 6.0 database. The results revealed that both LytS and CpsE were recognized as potential novel drug targets, as no known compounds targeting these proteins were found within the DrugBank database.

### Prediction and assessment of three-dimensional structures

**Fig 5:**
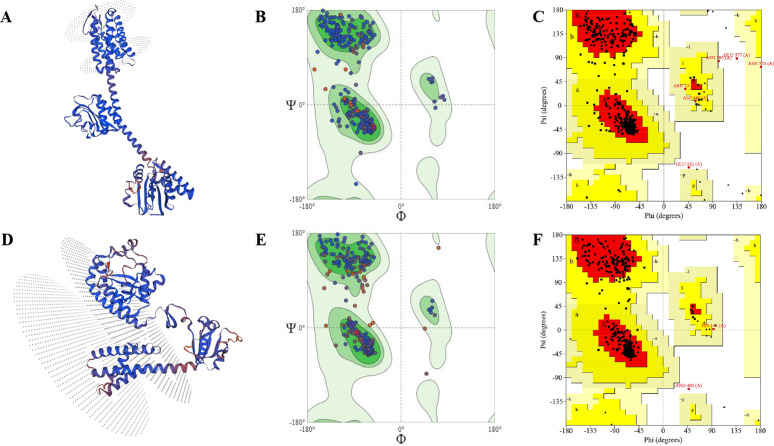
Determination of the three-dimensional structures. **(A)** Three-dimensional structure of Sensor protein LytS with Ramachandran plots generated by **(B)** Swiss-Model Structure Validation and **(C)** PROCHECK. **(D)** Three-dimensional structure of Galactosyl transferase CpsE with Ramachandran plots generated by **(B)** Swiss-Model Structure Validation and **(C)** PROCHECK.

**Fig 6:**
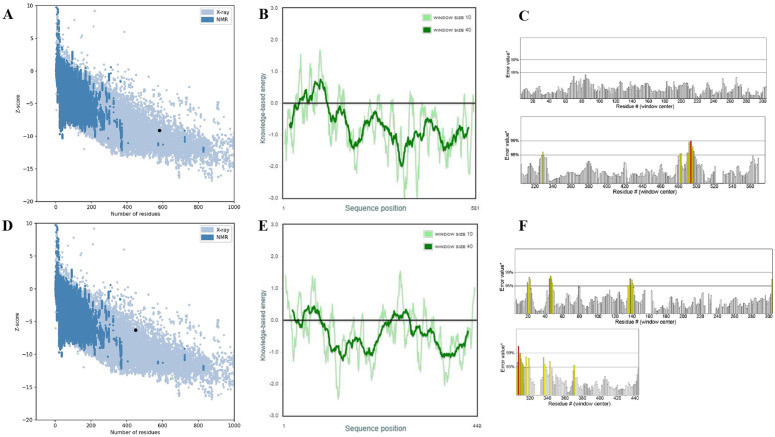
Structural quality assessment of the two target protein structures. **(A)** Overall quality assessment, (**B**) local quality assessment, and **(C)** ERRAT scores of the Sensor protein LytS structure. **(D)** Overall quality assessment, (**E**) local quality assessment, and **(F)** ERRAT scores of the Galactosyl transferase CpsE structure. Panels (**A**) and (**D**) display Z-scores representing the compatibility of the predicted 3D structures with known protein models of similar sizes. Panels (**B**) and (**E**) show per-residue knowledge-based energy profiles highlighting potential structural inaccuracies. Panels (**C**) and (**F**) present ERRAT scores evaluating model reliability based on non-bonded atom-atom interactions, where higher scores indicate better quality.

Understanding protein functions, ligand interactions, and dynamic behaviors relies heavily on the prediction of protein tertiary structures [103]. Thus, the online server Swiss-Model generated fifty initial templates for each protein, and the templates with the most GMQE score (LytS = 0.89, CpsE = 0.83), coverage (LytS = 1.00, CpsE = 1.00), sequence similarity (LytS = 0.55, CpsE = 0.61), range (LytS = 1–581, CpsE = 1–449) and identity percentage (LytS = 81.9, CpsE = 99.55) were chosen to construct the 3D models. Moreover, the predicted structures of Sensor protein LytS and Galactosyl transferase CpsE had 0.80 and 1.06 MolProbity scores, clash scores of 0.65 and 0.93, with 97.58% and 95.97% of residues in the favored regions of the Ramachandran plot respectively, according to the Swiss-Model structure validation (Fig 5). In addition, PROCHECK analysis revealed that the residues in the favored region of the Ramachandran plot of LytS and CpsE was 92.1% and 93.3%, respectively (Fig 5). The ERRAT scores of 98.025 for LytS and 93.253 for CpsE (Fig 6) further confirmed the good quality of the structures. Furthermore, the Z-scores of the proteins were −9.1 for LytS and −6.29 for CpsE (Fig 6). The accuracy and quality of the constructed 3D structures for the selected proteins were confirmed, suggesting that the predicted models conform to the typical structural range observed in NMR and X-ray crystallography data.

### Active site analysis

**Fig 7:**
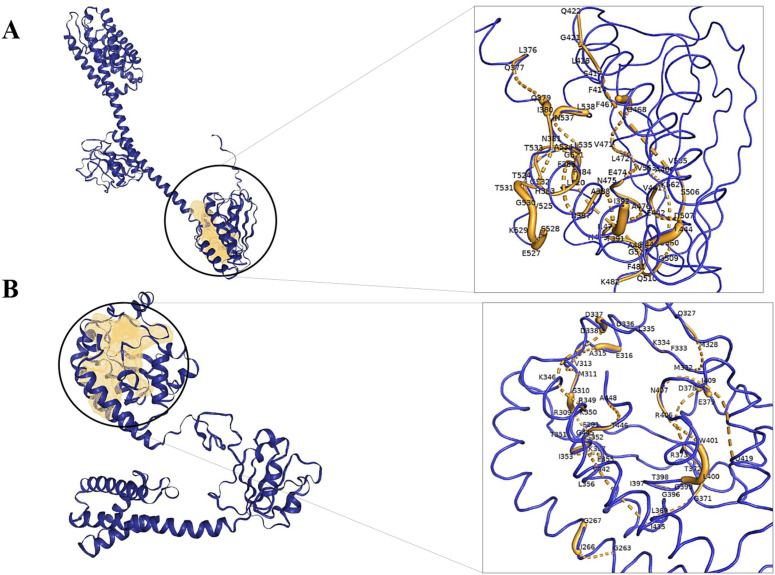
Active sites of the two target proteins. The active sites of the two target proteins, **(A)** Sensor protein LytS and **(B)** Galactosyl transferase CpsE, are highlighted in yellow, their corresponding amino acid residues are magnified in the inset, and their protein backbones are depicted as blue ribbons.

After modeling the protein structures, it was essential to identify a suitable binding interface to facilitate ligand binding. The DoGSiteScorer tool subsequently identified 19 potential binding pockets for the Sensor protein LytS, and the site exhibiting the highest drug score of 0.81 was favored. In a similar manner, 19 potential binding pockets were identified for Galactosyl transferase CpsE, and the pocket with the highest drug score of 0.81 was favored for additional analysis. S4 Table provides detailed information on the amino acid residues within the selected binding pockets of both LytS and CpsE, while the binding sites of both proteins are visually represented in Fig 7.

### Identification of ligands

**Fig 8:**
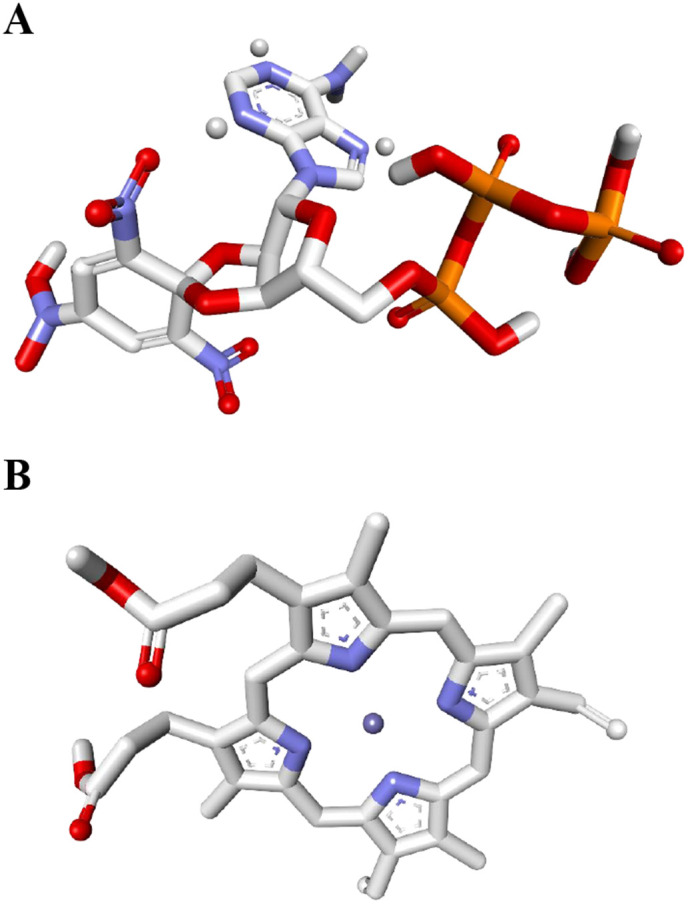
Ligands identified for the two selected drug targets. **(A)** Spiro(2,4,6-trinitrobenzene[1,2A]-2O’,3O’-methylene-adenine-triphosphate (Ligand 128) with assigned IUPAC name (3aR,4R,6R,6aR)-4-(6-aminopurin-9-yl)-N-hydroxy-6-[[hydroxy-[hydroxy(phosphonooxy)phosphoryl]oxyphosphoryl]oxymethyl]-3’,5’-dinitrospiro[3a,4,6,6a-tetrahydrofuro[3,4-d][1,3]dioxole-2,4’-cyclohexa-2,5-diene]-1’-imine oxide for Sensor protein LytS **(B)** Protoporphyrin-IX-containing-Fe (HEM) with IUPAC name 3-[18-(2-carboxyethyl)-8,13-bis(ethenyl)-3,7,12,17-tetramethyl-23H-porphyrin-21-id-2-yl]propanoate;iron(2+) for Galactosyl transferase CpsE. The nitrogen atoms of the ligands are depicted in blue, oxygen atoms in red, phosphorus atoms in orange, iron atoms in purple, and carbon atoms in white.

Protein binding site identification and their respective ligands plays a critical role in drug discovery and pharmaceutical research. These sites are crucial both structurally and functionally, serving as regions where different drug molecules bind to induce the desired biological response [93]. Exploiting the ProBis server, the ligand 128, officially named as Spiro(2,4,6-trinitrobenzene[1,2A]-2O’,3O’-methylene-adenine-triphosphate with the IUPAC designation (3*aR*,4*R*,6*R*,6*aR*)-4-(6-aminopurin-9-yl)-*N*-hydroxy-6-[[hydroxy-[hydroxy(phosphonooxy)phosphoryl]oxyphosphoryl]oxymethyl]-3’,5’-dinitrospiro[3*a*,4,6,6*a*-tetrahydrofuro[3,4-d][1,3]dioxole-2,4’-cyclohexa-2,5-diene]-1’-imine oxide, was identified in connection with the Sensor protein LytS. The ligand 128 was attained from a template with the PDB ID: 1I5D (from *Thermotoga maritima*). Whereas for Galactosyl transferase CpsE, the ligand HEM, formally named as Protoporphyrin-IX-containing-Fe with the IUPAC designation 3-[18-(2-carboxyethyl)-8,13-bis(ethenyl)-3,7,12,17-tetramethyl-23*H*-porphyrin-21-id-2-yl]propanoate;iron(2+), was attained from a template with the PDB ID: 2WDQ (from *Escherichia coli*). The structural representations of the two identified ligands are presented in Fig 8.

### Molecular docking studies

**Fig 9:**
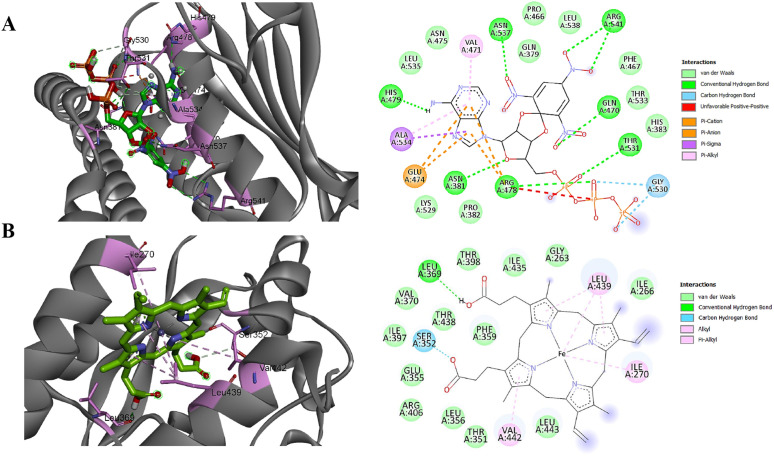
Illustration of the interactions between ligands and their corresponding drug targets through molecular docking. **(A)** Three-dimensional and two-dimensional interaction profiles of ligand 128 with the sensor protein LytS. **(B)** Three-dimensional and two-dimensional interaction profiles of ligand HEM with galactosyltransferase CpsE. In the three-dimensional representations, amino acid residues of the target proteins are shown as pink sticks, while interacting ligands are depicted in green, blue, and red. In the two-dimensional illustrations, dotted lines colored green, blue, pink, purple, and red indicate various types of bonds formed between the target proteins and their respective ligands.

The ligand exhibiting the lowest docking score in molecular docking studies is regarded as the most effective, as it signifies a stronger and more stable binding affinity with the target protein, thereby making it a prime candidate for further exploration in drug design and therapeutic development [104]. The interactions between the modeled structures of LytS and CpsE and their respective ligands were investigated using molecular docking performed with AutoDock Vina in PyRx v0.8. Ligand 128 demonstrated a stronger binding affinity with LytS, yielding a binding energy of –9.1 kcal/mol, whereas ligand HEM interacted with CpsE yielding a binding energy of –8.9 kcal/mol. As depicted in Fig 9, ligand 128 established hydrogen bonds with the amino acid residues His479, Asn537, Arg541, Gln470, Thr531, Arg478, and Asn381 of LytS. In comparison, ligand HEM interacted with CpsE through hydrogen bonds with the residues Ser352 and Leu369.

## Discussion

Developing new therapeutic drugs and vaccines are challenging. Advancements in computational research, sequence-based technologies, and the availability of diverse pathogens’ genomes and proteomics data have made it easier to perform the task. Subtractive genomics approach is invaluable for drug discovery because it isolates pathogen-specific proteins, minimizing potential harm to the host by targeting only the pathogen’s essential proteins. This method reduces the risk of off-target effects, ensuring therapeutic specificity. Non-homologous proteins differ from host proteins in that they do not share sequence similarity or functional characteristics, making them ideal candidates for drug targeting and vaccine development without interfering with the host’s own cellular machinery. *In silico* subtractive genome techniques are promising in identifying specific genes and proteins in various organisms including *Clostridium botulinum* [105]*, Mycoplasma pneumoniae* [55]*, Streptococcus pneumonia* [106], *Campylobacter jejuni* [107]*, Salmonella typhi* [50], *Legionella pneumophila* [63], *Arabidopsis thaliana* [108], *Meningococcus B* [109]*, Eubacterium notadum* [110], *Fusobacterium nucleatum* [111], *Salmonella enterica subsp. Poona* [112], *Treponema pallidum* [113], *Staphylococcus aureus N315* [114], *Acinetobacter baumanii* [115,116], *Bartonella bacilliformis* [117], *Bordetella pertussis* [118], *Serratia marcescens* [119], and *Staphylococcus aureus* [120].

In this study, we employed a subtractive genomics-based computational approach to screen the entire proteome of *S. agalactiae* serotype V (ATCC BAA-611/ 2603 V/R) for the identification of potential drug targets. Afterwards, 200 proteins from the set of peer-reviewed proteins of the pathogen were recognized to have no similarities to the host *H. sapiens* proteins. In some studies, human homologous proteins (up to 40% similarity) were selected to identify potential drug targets [121], based on the idea that low similarity in sequence would produce a slightly different protein structure from human host and it would be safely considered as a drug target [121,122]. This could be the case, when no potential drug targets are found in the non-homologous sequence pool. Later on, we utilized the DEG database version 15.2, that successfully lead to the selection of essential non-similar proteins out of the non-homologous sequences (S1 Table). The promising candidates for organism-specific drug targets are proteins that are vital for the pathogen’s survival and do not share homology with the host genome [72,98,115,117,123].

Subsequently, KEGG KAAS analysis revealed 51 essential non-homologous proteins were involved in 41 unique pathways of the pathogen (Table 2). These unique pathways including two-component system, quorum sensing, peptidoglycan biosynthesis, and various amino acid metabolism systems such as alanine, aspartate, glutamate, cysteine, methionine, tyrosine, histidine metabolism, etc., are essential for the survival of *S. agalactiae* and when interrupted would cause the bacterium to not function properly [54,107]. Environmental stress can increase gene expression and metabolic processes in bacteria, potentially leading to the development of resistant pathogens with distinct metabolic processes [62,120]. Thus, these essential proteins involved in such unique pathways alone could be excellent targets for drugs and vaccines [62,120].

In addition, protein localization is crucial in drug development, as it governs the formulation and design of novel drugs and vaccines, as demonstrated in studies including *Acinetobacter baumanii* [46,115], *Bartonella bacilliformis* [117], *Bordetella pertussis* [118], *Streptococcus pneumoniae* [57,124], *Salmonella typhi* [48], *Mycoplasma genitalium* [125], *Neisseria meningitides* [60], *Staphylococcus aureus* [62,120], and *Treponema pallidum* [113]. In several studies, cytoplasmic proteins were selected as drug targets for their availability and involvement in metabolic pathways crucial to the survival of those pathogens [44,58,125,126]. However, in our study we emphasized on the cytoplasmic membrane proteins in view of the fact that they can further be utilized as a target for vaccine development, as membrane proteins are considered to be the best criteria for vaccines [106,127,128]. Moreover, it was seen that membrane proteins are essential for integral cellular signal detection, transduction, and various other biological processes [129]. They act as diffusion barriers for ions, water, transport systems, and nutrients and when disrupted or degraded, can lead the bacteria to cease functioning and can serve as excellent druggable targets [129]. It is important to note that a significant number of druggable targets were derived from membrane proteins [45,127–135].

In addition, the identification of unique virulent factors of *S. agalactiae* would represent a substantial contribution, given that these factors are crucial in the control or degradation of the host immune system [136]. In *Helicobacter pylori*, virulence factors like VacA, a vacuolating cytotoxin, are associated with the development of gastric diseases, with specific genotypes linked to ulcerogenic and non-ulcerogenic strains [137]. Hence, from the set of essential proteins, six virulent proteins (Table 4) were predicted by the tool VirulentPred2.0, implying that inhibition of these proteins could render the pathogen non-virulent and significantly impact the pathogen’s functionality within the host organism [47,49,50,59,105,111]. As a result, we prioritized two proteins, namely sensor protein LytS and galactosyl transferase CpsE, that had the potential to be utilized as promising drug candidates because of their virulent nature, cytoplasmic membrane characteristics and their involvement in essential pathogen-specific pathways of *S. agalactiae*.

The sensor protein LytS, also known as sensor histidine kinase LytS, encoded by the gene *lytS*, is a part of the two-component regulatory system LytR/LytS of *S. agalactiae*. Two-component regulatory systems are key contributors to bacterial responses to environmental changes, regulating gene expression in response to stimuli, which enables bacteria to perceive, respond, and cope with stressful conditions [138,139]. LytS, self-phosphorylates its cytoplasmic domain by transferring the phosphate group upon detecting specific signals or stress conditions, activating the transcriptional response regulator LytR. The phosphorylated LytR then binds to DNA, regulating the expression of target genes [138,139]. The LytSR two-component system, consequently, influences virulence factors like biofilm formation, resistance to host antimicrobial peptides, and bacterial autolysis, enhancing the survival of *S. agalactiae* in the human host [22,138,139]. In essence, LytS acts as a molecular switch, initiating a signaling cascade that influences gene expression, behavior, and virulence, enhancing bacterial adaptation and making it a key player in pathogenesis. Disruption of LytS would impair the bacterial ability to respond to environmental stresses, reduce cell wall integrity, and prevent the activation of virulence factors regulated by the two-component system.

Conversely, galactosyl transferase CpsE, encoded by the gene *cpsE*, is a key enzyme in the biosynthesis of capsular polysaccharide and played a pivotal role in the assemblage of capsular polysaccharide in group B streptococci [32,140]. The polysaccharide capsule, consisting of a series of repeating monosaccharides units including glucose, galactose, and N-acetylglucosamine, represents a significant virulence factor in pathogens belonging to the *Streptococcus* genus and plays a significant role in enabling these bacteria to evade the innate immune response by providing protection against phagocytosis, opsonization, along with the complement system [32,141]. CpsE catalyzes the transfer of a galactose residue from UDP-galactose (UDP-Gal) to an undecaprenyl phosphate acceptor, initiating the formation of the CPS oligosaccharide repeating unit, which occurs at the cytoplasmic face of the bacterial cell wall [32]. CpsE is part of a larger biosynthetic complex, which includes other enzymes like CpsJ, CpsK, and CpsA, each playing distinct roles in capsular polymerization, sialylation, and insertion into the bacterial cell wall [32]. Moreover, CPS biosynthesis influences biofilm formation, further enhancing GBS pathogenicity during infection. Thus, inhibiting CpsE weakens the protective capsule, exposing the bacteria to phagocytosis and immune detection, thereby increasing sensitivity to treatment and immune responses.

The STRING server afterwards revealed that the two selected proteins could function as core proteins associating with three or more neighboring proteins of *S. agalactiae*. As a result, repressing these proteins can inhibit the proper functioning of other related proteins [56,58,63,119]. ProtParam analysis predicted that both proteins were hydrophobic, basic, stable, and thermostable in nature, with LytS having 581 amino acids and CpsE having 449 amino acids in length (Table 5). Furthermore, druggability analysis revealed both proteins to be novel drug targets.

In addition, the study effectively predicted, analyzed, and evaluated the 3D structures of the two chosen proteins. It was seen that the residues in the Ramachandran favoured regions of both proteins were more than 85% and the Molprobity score was between the expected range of −4–2 [142], thereby confirming that the above values and calculations satisfied the structural validation criteria and showed that the predicted structures were of high quality as reported in various other studies [44,49,50,56,58,59,61,63,108,110,112,113,119,120,143,144]. Moreover, through the utilization of the ProBis server, ligand 128 was identified as a potential binder for LytS, while HEM was found to interact with CpsE. The binding sites of both proteins were analyzed through the DoGSiteScorer tool and visualized using PyMOL 3.1. Subsequent molecular docking studies revealed that both ligands exhibited notable binding affinities. Specifically, ligand 128 demonstrated a binding affinity of –9.1 kcal/mol with LytS, while HEM displayed a binding affinity of –8.9 kcal/mol with CpsE. These findings underscore the significant binding interactions between the ligands and their targets, suggesting their potential utility in the modulation of these proteins for therapeutic applications. As a result, we successfully identified and analyzed new proteins that show great promise as potential therapeutic targets. The potential of these proteins was prudently determined pertaining to their fundamental contribution to play an essential part in the survival of the pathogen and their feasibility in combating *S. agalactiae* serotype V (Strain ATCC BAA-611/2603V/R). As far as we are aware, this serotype has never been the subject of a subtractive genomics study before, and we believe it will provide a promising new strategy for preventing the spread of the antibiotic-resistant bacteria. Furthermore, to evaluate the potential for cross-species drug targeting, we performed a BLASTp-based sequence homology analysis of *S. agalactiae* serotype V proteins LytS and CpsE against a panel of clinically relevant bacterial pathogens. These proteins exhibited a range of 40% to 98% sequence similarity to various sensor histidine kinases and sugar transferases across multiple species, including *Bacillus anthracis*, *Streptococcus pneumoniae*, *Staphylococcus aureus*, and *Mycobacterium tuberculosis*, among others (S5 Table). High sequence identity (>70%), as observed with the LytS homolog in *S. mutans*, suggests conserved structural domains and potential drug-binding sites, supporting the feasibility of multitarget antimicrobial development. Proteins with moderate similarity (40–60%), such as homologs in *S. pneumoniae*, *B. subtilis*, and *C. botulinum*, may still serve as viable targets, pending structural validation of conserved ligand-binding pockets. In contrast, homologs with <40% identity are less likely to be druggable unless functionally conserved domains are retained. However, it requires further structural and functional investigations which is not in the scope of the current article.

## Conclusion

The development of new drugs has been significantly expedited by the utilization of bioinformatics tools to extract and analyze genome and proteome sequences from diverse pathogens across multiple databases. The subtractive genomics methodology is impressively capable of effectively resolving challenges which are prevalent in traditional drug discovery methodologies. Using subtractive genomics approach, we have successfully identified two proteins, namely LytS and CpsE, that have the potential to be used as drug targets. While this research establishes a strong computational foundation in early-stage drug discovery, it also opens avenues for further *in vitro* and *in vivo* studies that are crucial for translating these computational insights into clinically viable therapies, which is beyond the scope of the current work. Nonetheless, an extensive pipeline for drug target identification has been developed in this study, which has the potential to facilitate and aid future experimental research to functionally characterize these targets *in vivo* and validate their druggability through high-throughput screening and preclinical evaluation. This approach holds promise for the creation of alternative therapies, offering a valuable framework for future drug discovery.

## Supporting information

S1 Table All DEG Essential Proteins. (XLSX)

S2 Table List of KO ID. (XLSX)

S3 Table All Subcellular Localizations. (XLSX)

S4 Table Residues present in the active sites of Sensor protein LytS. (XLSX)

S5 Table Galactosyl transferase CpsE. (XLSX)

## References

[1] Whiley RA, Hardie JM. Systematic Bacteriology. 2nd Edition. New York, NY: Springer New York; 2009. doi: 10.1007/978-0-387-68489-5

[2] Ray C, Ryan KJ. Sherris Medical Microbiology: An Introduction to Infectious Diseases. 4th ed. McGraw-Hill Medical. 2003.

[3] Ferrieri P, Blair LL. Pharyngeal carriage of group B streptococci: detection by three methods. J Clin Microbiol. 1977;6(2):136–9. doi: 10.1128/jcm.6.2.136-139.1977 330561 PMC274720

[4] van der Mee-Marquet N, Fourny L, Arnault L, Domelier A-S, Salloum M, Lartigue M-F, et al. Molecular characterization of human-colonizing *Streptococcus agalactiae* strains isolated from throat, skin, anal margin, and genital body sites. J Clin Microbiol. 2008;46(9):2906–11. doi: 10.1128/JCM.00421-08 18632904 PMC2546740

[5] Armistead B, Oler E, Adams Waldorf K, Rajagopal L. The Double Life of Group B Streptococcus: Asymptomatic Colonizer and Potent Pathogen. J Mol Biol. 2019;431(16):2914–31. doi: 10.1016/j.jmb.2019.01.035 30711542 PMC6646060

[6] Delfani S, Bahmani M, Mohammadrezaei-Khorramabadi R, Rafieian-Kopaei M. Phytotherapy in *Streptococcus agalactiae*: An Overview of the Medicinal Plants Effective against *Streptococcus agalactiae*. J Clin Diagn Res. 2017;11(6):DE01–2. doi: 10.7860/JCDR/2017/25530.9988 28764166 PMC5535359

[7] Keefe GP. *Streptococcus agalactiae* mastitis: a review. Can Vet J. 1997;38(7):429–37. 9220132 PMC1576741

[8] Slotved H-C, Kong F, Lambertsen L, Sauer S, Gilbert GL. Serotype IX, a Proposed New *Streptococcus agalactiae* Serotype. J Clin Microbiol. 2007;45(9):2929–36. doi: 10.1128/JCM.00117-07 17634306 PMC2045254

[9] Low DE. Nonpneumococcal StreptoCOccal Infections, Rheumatic Fever. Goldman’s Cecil Medicine. Elsevier. 2012:1823–9. doi: 10.1016/b978-1-4377-1604-7.00298-0

[10] Johri AK, Paoletti LC, Glaser P, Dua M, Sharma PK, Grandi G, et al. Group B Streptococcus: global incidence and vaccine development. Nat Rev Microbiol. 2006;4(12):932–42. doi: 10.1038/nrmicro1552 17088932 PMC2742968

[11] Lancefield RC, Hare R. The serological differentiation of pathogenic and non-pathogenic strains of hemolytic streptococci from parturient women. J Exp Med. 1935;61(3):335–49. doi: 10.1084/jem.61.3.335 19870362 PMC2133228

[12] Eickhoff TC, Klein JO, Daly AK, Ingall D, Finland M. Neonatal sepsis and other infections due to group b beta-hemolytic streptococci. N Engl J Med. 1964;271:1221–8. doi: 10.1056/NEJM196412102712401 14234266

[13] Phares CR, Lynfield R, Farley MM, Mohle-Boetani J, Harrison LH, Petit S, et al. Epidemiology of invasive group B streptococcal disease in the United States, 1999-2005. JAMA. 2008;299(17):2056–65. doi: 10.1001/jama.299.17.2056 18460666

[14] Brouwer MC, Tunkel AR, van de Beek D. Epidemiology, diagnosis, and antimicrobial treatment of acute bacterial meningitis. Clin Microbiol Rev. 2010;23(3):467–92. doi: 10.1128/CMR.00070-09 20610819 PMC2901656

[15] Le Doare K, Heath PT. An overview of global GBS epidemiology. Vaccine. 2013;31 Suppl 4:D7–12. doi: 10.1016/j.vaccine.2013.01.009 23973349

[16] Koenig JM, Keenan WJ. Group B streptococcus and early-onset sepsis in the era of maternal prophylaxis. Pediatr Clin North Am. 2009;56(3):689–708, Table of Contents. doi: 10.1016/j.pcl.2009.04.003 19501699 PMC2702484

[17] Murray PR, Baron EJ, Jorgensen JH, Landry ML, Pfaller MA. Manual of Clinical Microbiology. 9th ed. Washington: ASM Press. 2007.

[18] Kothari NJ, Morin CA, Glennen A, Jackson D, Harper J, Schrag SJ, et al. Invasive group B streptococcal disease in the elderly, Minnesota, USA, 2003-2007. Emerg Infect Dis. 2009;15(8):1279–81. doi: 10.3201/eid1508.081381 19751591 PMC2815956

[19] Seale AC, Bianchi-Jassir F, Russell NJ, Kohli-Lynch M, Tann CJ, Hall J, et al. Estimates of the Burden of Group B Streptococcal Disease Worldwide for Pregnant Women, Stillbirths, and Children. Clin Infect Dis. 2017;65(suppl_2):S200–19. doi: 10.1093/cid/cix664 29117332 PMC5849940

[20] Raabe VN, Shane AL. Group B *Streptococcus* (*Streptococcus agalactiae*). Microbiol Spectr. 2019;7(2). doi: 10.1128/microbiolspec.GPP3-0007-2018 30900541 PMC6432937

[21] A’Hearn-Thomas B, Khatami A, Randis TM, Vurayai M, Mokomane M, Arscott-Mills T, et al. High Rate of Serotype V *Streptococcus agalactiae* Carriage in Pregnant Women in Botswana. The American Journal of Tropical Medicine and Hygiene. 2019;100(5):1115–7. doi: 10.4269/ajtmh.18-0847 30915949 PMC6493924

[22] Shabayek S, Spellerberg B. Group B Streptococcal Colonization, Molecular Characteristics, and Epidemiology. Front Microbiol. 2018;9. doi: 10.3389/fmicb.2018.00437 PMC5861770 29593684

[23] Bergal A, Loucif L, Benouareth DE, Bentorki AA, Abat C, Rolain J-M. Molecular epidemiology and distribution of serotypes, genotypes, and antibiotic resistance genes of *Streptococcus agalactiae* clinical isolates from Guelma, Algeria and Marseille, France. Eur J Clin Microbiol Infect Dis. 2015;34(12):2339–48. doi: 10.1007/s10096-015-2487-6 26415872

[24] Gizachew M, Tiruneh M, Moges F, Tessema B. *Streptococcus agalactiae* maternal colonization, antibiotic resistance and serotype profiles in Africa: a meta-analysis. Ann Clin Microbiol Antimicrob. 2019;18(1):14. doi: 10.1186/s12941-019-0313-1 30922308 PMC6437888

[25] Pinto AM, Pereira TA, Alves V, Araújo A, Lage OM. Incidence and serotype characterisation of *Streptococcus agalactiae* in a Portuguese hospital. J Clin Pathol. 2018;71(6):508–13. doi: 10.1136/jclinpath-2017-204646 29180508

[26] Dutra VG, Alves VMN, Olendzki AN, Dias CAG, de Bastos AFA, Santos GO, et al. *Streptococcus agalactiae* in Brazil: serotype distribution, virulence determinants and antimicrobial susceptibility. BMC Infect Dis. 2014;14:323. doi: 10.1186/1471-2334-14-323 24919844 PMC4061772

[27] do Nascimento CS, Dos Santos NFB, Ferreira RCC, Taddei CR. *Streptococcus agalactiae* in pregnant women in Brazil: prevalence, serotypes, and antibiotic resistance. Braz J Microbiol. 2019;50(4):943–52. doi: 10.1007/s42770-019-00129-8 31432465 PMC6863207

[28] Tulyaprawat O, Pharkjaksu S, Shrestha RK, Ngamskulrungroj P. Emergence of Multi-Drug Resistance and Its Association With Uncommon Serotypes of *Streptococcus agalactiae* Isolated From Non-neonatal Patients in Thailand. Front Microbiol. 2021;12:719353. doi: 10.3389/fmicb.2021.719353 34566923 PMC8456118

[29] Genovese C, D’Angeli F, Di Salvatore V, Tempera G, Nicolosi D. *Streptococcus agalactiae* in pregnant women: serotype and antimicrobial susceptibility patterns over five years in Eastern Sicily (Italy). Eur J Clin Microbiol Infect Dis. 2020;39(12):2387–96. doi: 10.1007/s10096-020-03992-8 32700131 PMC7669783

[30] Kaminska D, Ratajczak M, Szumała-Kąkol A, Dlugaszewska J, Nowak-Malczewska DM, Gajecka M. Increasing Resistance and Changes in Distribution of Serotypes of *Streptococcus agalactiae* in Poland. Pathogens. 2020;9(7):526. doi: 10.3390/pathogens9070526 32610654 PMC7400139

[31] Founou LL, Khan UB, Medugu N, Pinto TCA, Darboe S, Chendi Z, et al. Molecular epidemiology of *Streptococcus agalactiae* in non-pregnant populations: a systematic review. Microb Genom. 2023;9(11):001140. doi: 10.1099/mgen.0.001140 38019122 PMC10711312

[32] Noble K, Lu J, Guevara MA, Doster RS, Chambers SA, Rogers LM, et al. Group B Streptococcus cpsE Is Required for Serotype V Capsule Production and Aids in Biofilm Formation and Ascending Infection of the Reproductive Tract during Pregnancy. ACS Infect Dis. 2021;7(9):2686–96. doi: 10.1021/acsinfecdis.1c00182 34076405 PMC8588567

[33] Rosini R, Margarit I. Biofilm formation by *Streptococcus agalactiae*: influence of environmental conditions and implicated virulence factors. Front Cell Infect Microbiol. 2015;5:6. doi: 10.3389/fcimb.2015.00006 25699242 PMC4316791

[34] Hsu C-Y, Moradkasani S, Suliman M, Uthirapathy S, Zwamel AH, Hjazi A, et al. Global patterns of antibiotic resistance in group B Streptococcus: a systematic review and meta-analysis. Front Microbiol. 2025;16:1541524. doi: 10.3389/fmicb.2025.1541524 40342597 PMC12060732

[35] Zakerifar M, Kaboosi H, Goli HR, Rahmani Z, Peyravii Ghadikolaii F. Antibiotic resistance genes and molecular typing of *Streptococcus agalactiae* isolated from pregnant women. BMC Pregnancy Childbirth. 2023;23(1):43. doi: 10.1186/s12884-023-05380-4 36658541 PMC9854082

[36] Abbasi Montazeri E, Seyed-Mohammadi S, Asarehzadegan Dezfuli A, Khosravi AD, Dastoorpoor M, Roointan M, et al. Investigation of SCCmec types I-IV in clinical isolates of methicillin-resistant coagulase-negative staphylococci in Ahvaz, Southwest Iran. Biosci Rep. 2020;40(5):BSR20200847. doi: 10.1042/BSR20200847 32347308 PMC7214399

[37] Saleh RO, Al-Ouqaili MTS, Ali E, Alhajlah S, Kareem AH, Shakir MN, et al. lncRNA-microRNA axis in cancer drug resistance: particular focus on signaling pathways. Med Oncol. 2024;41(2):52. doi: 10.1007/s12032-023-02263-8 38195957

[38] Khoshnood S, Shahi F, Jomehzadeh N, Montazeri EA, Saki M, Mortazavi SM, et al. Distribution of genes encoding resistance to macrolides, lincosamides, and streptogramins among methicillin-resistant Staphylococcus aureus strains isolated from burn patients. Acta Microbiol Immunol Hung. 2019;66(3):387–98. doi: 10.1556/030.66.2019.015 31096760

[39] Leghari A, Lakho SA, Khand FM, Bhutto R, Lone SQ, Aleem MT, et al. Molecular epidemiology, characterization of virulence factors and antibiotic resistance profile of *Streptococcus agalactiae* isolated from dairy farms in China and Pakistan. Journal of Integrative Agriculture. 2023;22(5):1514–28. doi: 10.1016/j.jia.2022.10.004

[40] Kimura K, Suzuki S, Wachino J, Kurokawa H, Yamane K, Shibata N, et al. First molecular characterization of group B streptococci with reduced penicillin susceptibility. Antimicrob Agents Chemother. 2008;52(8):2890–7. doi: 10.1128/AAC.00185-08 18490507 PMC2493108

[41] Hayes K, O’Halloran F, Cotter L. A review of antibiotic resistance in Group B Streptococcus: the story so far. Crit Rev Microbiol. 2020;46(3):253–69. doi: 10.1080/1040841X.2020.1758626 32363979

[42] van der Linden M, Mamede R, Levina N, Helwig P, Vila-Cerqueira P, Carriço JA, et al. Heterogeneity of penicillin-non-susceptible group B streptococci isolated from a single patient in Germany. J Antimicrobial Chemotherapy. 2019;75(2):296–9. doi: 10.1093/jac/dkz465 PMC6966095 31740946

[43] Han G, Zhang B, Luo Z, Lu B, Luo Z, Zhang J, et al. Molecular typing and prevalence of antibiotic resistance and virulence genes in *Streptococcus agalactiae* isolated from Chinese dairy cows with clinical mastitis. PLoS ONE. 2022;17(5):e0268262. doi: 10.1371/journal.pone.0268262 PMC9075616 35522690

[44] Saleem H, Ashfaq UA, Nadeem H, Zubair M, Siddique MH, Rasul I. Subtractive genomics and molecular docking approach to identify drug targets against Stenotrophomonas maltophilia. PLoS ONE. 2021;16(12):e0261111. doi: 10.1371/journal.pone.0261111 PMC8673605 34910751

[45] Mohammed Neema K. In silico identification and characterization of novel drug targets and outer membrane proteins in the fish pathogen Edwardsiella tarda. OAB. 2011;37. doi: 10.2147/oab.s15581

[46] Goyal M, Citu C, Singh N. In silico identification of novel drug targets in acinetobacter baumannii by subtractive genomic approach. Asian J Pharm Clin Res. 2018;11(3):230. doi: 10.22159/ajpcr.2018.v11i3.22105

[47] Prabha R, Singh DP, Ahmad K, Kumar SPJ, Kumar P. Subtractive genomics approach for identification of putative antimicrobial targets in Xanthomonas oryzae pv. oryzae KACC10331. Archives of Phytopathology and Plant Protection. 2019;52(7–8):863–72. doi: 10.1080/03235408.2018.1562674

[48] Rathi B, Sarangi AN, Trivedi N. Genome subtraction for novel target definition in Salmonella typhi. Bioinformation. 2009;4(4):143–50. doi: 10.6026/97320630004143 20198190 PMC2825597

[49] Khan K, Uddin R. Integrated bioinformatics based subtractive genomics approach to decipher the therapeutic function of hypothetical proteins from Salmonella typhi XDR H-58 strain. Biotechnol Lett. 2022;44(2):279–98. doi: 10.1007/s10529-021-03219-6 35037232 PMC8761513

[50] Khan K, Jalal K, Uddin R. An integrated in silico based subtractive genomics and reverse vaccinology approach for the identification of novel vaccine candidate and chimeric vaccine against XDR Salmonella typhi H58. Genomics. 2022;114(2):110301. doi: 10.1016/j.ygeno.2022.110301 35149170

[51] Sakharkar KR, Sakharkar MK, Chow VTK. A novel genomics approach for the identification of drug targets in pathogens, with special reference to Pseudomonas aeruginosa. In Silico Biol. 2004;4(3):355–60. doi: 10.3233/isb-00138 15724285

[52] Perumal D, Lim CS, Sakharkar KR, Sakharkar MK. Differential genome analyses of metabolic enzymes in Pseudomonas aeruginosa for drug target identification. In Silico Biol. 2007;7(4–5):453–65. 18391237

[53] Dutta A, Singh SK, Ghosh P, Mukherjee R, Mitter S, Bandyopadhyay D. In silico identification of potential therapeutic targets in the human pathogen Helicobacter pylori. In Silico Biol. 2006;6(1–2):43–7. 16789912

[54] Pasala C, Chilamakuri CSR, Katari SK, Nalamolu RM, Bitla AR, Umamaheswari A. An in silico study: Novel targets for potential drug and vaccine design against drug resistant H. pylori. Microb Pathog. 2018;122:156–61. doi: 10.1016/j.micpath.2018.05.037 29800696

[55] Singh Sarita GSK, Pant K. K GMK. Definition of potential targets in mycoplasma pneumoniae through subtractive genome analysis. J Antivir Antiretrovir. 2010;02(02). doi: 10.4172/jaa.1000020

[56] Hosen MI, Tanmoy AM, Mahbuba D-A, Salma U, Nazim M, Islam MT, et al. Application of a subtractive genomics approach for in silico identification and characterization of novel drug targets in Mycobacterium tuberculosis F11. Interdiscip Sci. 2014;6(1):48–56. doi: 10.1007/s12539-014-0188-y 24464704

[57] Wadood A, Jamal A, Riaz M, Khan A, Uddin R, Jelani M, et al. Subtractive genome analysis for in silico identification and characterization of novel drug targets in Streptococcus pneumonia strain JJA. Microb Pathog. 2018;115:194–8. doi: 10.1016/j.micpath.2017.12.063 29277475

[58] Khan K, Jalal K, Khan A, Al-Harrasi A, Uddin R. Comparative Metabolic Pathways Analysis and Subtractive Genomics Profiling to Prioritize Potential Drug Targets Against Streptococcus pneumoniae. Front Microbiol. 2022;12:796363. doi: 10.3389/fmicb.2021.796363 35222301 PMC8866961

[59] Omeershffudin UNM, Kumar S. Antibiotic resistance in Neisseria gonorrhoeae: broad-spectrum drug target identification using subtractive genomics. Genomics Inform. 2023;21(1):e5. doi: 10.5808/gi.22066 37037463 PMC10085745

[60] Narayan Sarangi A. Subtractive Genomics Approach for in Silico Identification and Characterization of Novel Drug Targets in Neisseria Meningitides Serogroup B. J Comput Sci Syst Biol. 2009;02(05). doi: 10.4172/jcsb.1000038

[61] Musharaf Hossain M, Mosnaz ATMJ, Sajib AM, Roy PK, Shakil SK, Sarid Ullah SM. Identification of putative drug targets of Listeria monocytogenes F2365 by subtractive genomics approach. J BioSci Biotech. 2013;63–71.

[62] Uddin R, Saeed K. Identification and characterization of potential drug targets by subtractive genome analyses of methicillin resistant Staphylococcus aureus. Comput Biol Chem. 2014;48:55–63. doi: 10.1016/j.compbiolchem.2013.11.005 24361957

[63] Khan MT, Mahmud A, Hasan M, Azim KF, Begum MK, Rolin MH, et al. Proteome Exploration of Legionella pneumophila To Identify Novel Therapeutics: a Hierarchical Subtractive Genomics and Reverse Vaccinology Approach. Microbiol Spectr. 2022;10(4):e0037322. doi: 10.1128/spectrum.00373-22 35863001 PMC9430848

[64] Thomas L, Cook L. Two-Component Signal Transduction Systems in the Human Pathogen *Streptococcus agalactiae*. Infect Immun. 2020;88(7):e00931–19. doi: 10.1128/IAI.00931-19 31988177 PMC7309623

[65] Apweiler R, Bairoch A, Wu CH, Barker WC, Boeckmann B, Ferro S, et al. UniProt: the Universal Protein knowledgebase. Nucleic Acids Res. 2004;32(Database issue):D115–9. doi: 10.1093/nar/gkh131 14681372 PMC308865

[66] UniProt Consortium T. UniProt: the universal protein knowledgebase. Nucleic Acids Res. 2018;46(5):2699. doi: 10.1093/nar/gky092 29425356 PMC5861450

[67] UniProt Consortium. UniProt: the Universal Protein Knowledgebase in 2023. Nucleic Acids Res. 2023;51(D1):D523–31. doi: 10.1093/nar/gkac1052 36408920 PMC9825514

[68] Altschul SF, Gish W, Miller W, Myers EW, Lipman DJ. Basic local alignment search tool. J Mol Biol. 1990;215(3):403–10. doi: 10.1016/S0022-2836(05)80360-2 2231712

[69] Zhang R, Ou H-Y, Zhang C-T. DEG: a database of essential genes. Nucleic Acids Res. 2004;32(Database issue):D271–2. doi: 10.1093/nar/gkh024 14681410 PMC308758

[70] Zhang R, Lin Y. DEG 5.0, a database of essential genes in both prokaryotes and eukaryotes. Nucleic Acids Res. 2009;37(Database issue):D455–8. doi: 10.1093/nar/gkn858 18974178 PMC2686491

[71] Luo H, Lin Y, Gao F, Zhang C-T, Zhang R. DEG 10, an update of the database of essential genes that includes both protein-coding genes and noncoding genomic elements. Nucleic Acids Res. 2014;42(Database issue):D574–80. doi: 10.1093/nar/gkt1131 24243843 PMC3965060

[72] Luo H, Lin Y, Liu T, Lai F-L, Zhang C-T, Gao F, et al. DEG 15, an update of the Database of Essential Genes that includes built-in analysis tools. Nucleic Acids Res. 2021;49(D1):D677–86. doi: 10.1093/nar/gkaa917 33095861 PMC7779065

[73] Moriya Y, Itoh M, Okuda S, Yoshizawa AC, Kanehisa M. KAAS: an automatic genome annotation and pathway reconstruction server. Nucleic Acids Res. 2007;35(Web Server issue):W182–5. doi: 10.1093/nar/gkm321 17526522 PMC1933193

[74] Yu NY, Wagner JR, Laird MR, Melli G, Rey S, Lo R, et al. PSORTb 3.0: improved protein subcellular localization prediction with refined localization subcategories and predictive capabilities for all prokaryotes. Bioinformatics. 2010;26(13):1608–15. doi: 10.1093/bioinformatics/btq249 20472543 PMC2887053

[75] Gardy JL, Laird MR, Chen F, Rey S, Walsh CJ, Ester M, et al. PSORTb v.2.0: expanded prediction of bacterial protein subcellular localization and insights gained from comparative proteome analysis. Bioinformatics. 2005;21(5):617–23. doi: 10.1093/bioinformatics/bti057 15501914

[76] Yu C-S, Lin C-J, Hwang J-K. Predicting subcellular localization of proteins for Gram-negative bacteria by support vector machines based on n-peptide compositions. Protein Sci. 2004;13(5):1402–6. doi: 10.1110/ps.03479604 15096640 PMC2286765

[77] Yu C-S, Chen Y-C, Lu C-H, Hwang J-K. Prediction of protein subcellular localization. Proteins. 2006;64(3):643–51. doi: 10.1002/prot.21018 16752418

[78] Garg A, Gupta D. VirulentPred: a SVM based prediction method for virulent proteins in bacterial pathogens. BMC Bioinformatics. 2008;9:62. doi: 10.1186/1471-2105-9-62 18226234 PMC2254373

[79] Gasteiger E, Hoogland C, Gattiker A, Duvaud S, Wilkins MR, Appel RD, et al. Protein Identification and Analysis Tools on the Expasy Server. In: Walker JM, editor. The Proteomics Protocols Handbook. Totowa, NJ: Humana Press; 2005:571–601. doi: 10.1385/1592598900

[80] Szklarczyk D, Gable AL, Lyon D, Junge A, Wyder S, Huerta-Cepas J, et al. STRING v11: protein-protein association networks with increased coverage, supporting functional discovery in genome-wide experimental datasets. Nucleic Acids Res. 2019;47(D1):D607–13. doi: 10.1093/nar/gky1131 30476243 PMC6323986

[81] Knox C, Wilson M, Klinger CM, Franklin M, Oler E, Wilson A, et al. DrugBank 6.0: the DrugBank Knowledgebase for 2024. Nucleic Acids Res. 2024;52(D1):D1265–75. doi: 10.1093/nar/gkad976 37953279 PMC10767804

[82] Arnold K, Bordoli L, Kopp J, Schwede T. The SWISS-MODEL workspace: a web-based environment for protein structure homology modelling. Bioinformatics. 2006;22(2):195–201. doi: 10.1093/bioinformatics/bti770 16301204

[83] Waterhouse A, Bertoni M, Bienert S, Studer G, Tauriello G, Gumienny R, et al. SWISS-MODEL: homology modelling of protein structures and complexes. Nucleic Acids Res. 2018;46(W1):W296–303. doi: 10.1093/nar/gky427 29788355 PMC6030848

[84] Guex N, Peitsch MC, Schwede T. Automated comparative protein structure modeling with SWISS-MODEL and Swiss-PdbViewer: a historical perspective. Electrophoresis. 2009;30 Suppl 1:S162–73. doi: 10.1002/elps.200900140 19517507

[85] Bienert S, Waterhouse A, de Beer TAP, Tauriello G, Studer G, Bordoli L, et al. The SWISS-MODEL Repository-new features and functionality. Nucleic Acids Res. 2017;45(D1):D313–9. doi: 10.1093/nar/gkw1132 27899672 PMC5210589

[86] Waterhouse AM, Studer G, Robin X, Bienert S, Tauriello G, Schwede T. The structure assessment web server: for proteins, complexes and more. Nucleic Acids Res. 2024;52(W1):W318–23. doi: 10.1093/nar/gkae270 38634802 PMC11223858

[87] Chen VB, Arendall WB 3rd, Headd JJ, Keedy DA, Immormino RM, Kapral GJ, et al. MolProbity: all-atom structure validation for macromolecular crystallography. Acta Crystallogr D Biol Crystallogr. 2010;66(Pt 1):12–21. doi: 10.1107/S0907444909042073 20057044 PMC2803126

[88] Laskowski RA, Rullmannn JA, MacArthur MW, Kaptein R, Thornton JM. AQUA and PROCHECK-NMR: programs for checking the quality of protein structures solved by NMR. J Biomol NMR. 1996;8(4):477–86. doi: 10.1007/BF00228148 9008363

[89] Laskowski RA, MacArthur MW, Moss DS, Thornton JM. PROCHECK: a program to check the stereochemical quality of protein structures. J Appl Crystallogr. 1993;26(2):283–91. doi: 10.1107/s0021889892009944

[90] Wiederstein M, Sippl MJ. ProSA-web: interactive web service for the recognition of errors in three-dimensional structures of proteins. Nucleic Acids Res. 2007;35(Web Server issue):W407–10. doi: 10.1093/nar/gkm290 17517781 PMC1933241

[91] Volkamer A, Griewel A, Grombacher T, Rarey M. Analyzing the topology of active sites: on the prediction of pockets and subpockets. J Chem Inf Model. 2010;50(11):2041–52. doi: 10.1021/ci100241y 20945875

[92] Volkamer A, Kuhn D, Grombacher T, Rippmann F, Rarey M. Combining global and local measures for structure-based druggability predictions. J Chem Inf Model. 2012;52(2):360–72. doi: 10.1021/ci200454v 22148551

[93] Konc J, Miller BT, Štular T, Lešnik S, Woodcock HL, Brooks BR, et al. ProBiS-CHARMMing: Web Interface for Prediction and Optimization of Ligands in Protein Binding Sites. J Chem Inf Model. 2015;55(11):2308–14. doi: 10.1021/acs.jcim.5b00534 26509288 PMC8725999

[94] Dallakyan S, Olson AJ. Small-molecule library screening by docking with PyRx. Methods Mol Biol. 2015;1263:243–50. doi: 10.1007/978-1-4939-2269-7_19 25618350

[95] Swango KL, Hymes J, Brown P, Wolf B. Amino acid homologies between human biotinidase and bacterial aliphatic amidases: putative identification of the active site of biotinidase. Mol Genet Metab. 2000;69(2):111–5. doi: 10.1006/mgme.2000.2959 10720437

[96] Hediger MA, Turk E, Wright EM. Homology of the human intestinal Na+/glucose and Escherichia coli Na+/proline cotransporters. Proc Natl Acad Sci U S A. 1989;86(15):5748–52. doi: 10.1073/pnas.86.15.5748 2490366 PMC297707

[97] Raman K, Yeturu K, Chandra N. targetTB: a target identification pipeline for Mycobacterium tuberculosis through an interactome, reactome and genome-scale structural analysis. BMC Syst Biol. 2008;2:109. doi: 10.1186/1752-0509-2-109 19099550 PMC2651862

[98] Ahmad S, Navid A, Akhtar AS, Azam SS, Wadood A, Pérez-Sánchez H. Subtractive genomics, molecular docking and molecular dynamics simulation revealed LpxC as a potential drug target against multi-drug resistant Klebsiella pneumoniae. Interdiscip Sci Comput Life Sci. 2018;11(3):508–26. doi: 10.1007/s12539-018-0299-y 29721784

[99] Schomburg D, Stephan D. Bis(5’-nucleosyl)-tetraphosphatase (symmetrical). Enzyme Handbook 16. Berlin, Heidelberg: Springer Berlin Heidelberg; 1998:671–675. doi: 10.1007/978-3-642-58903-4_133

[100] Casadevall A, Pirofski L-A. Virulence factors and their mechanisms of action: the view from a damage-response framework. J Water Health. 2009;7 Suppl 1:S2–18. doi: 10.2166/wh.2009.036 19717929

[101] Duan Q, Zhou M, Zhu L, Zhu G. Flagella and bacterial pathogenicity. J Basic Microbiol. 2013;53(1):1–8. doi: 10.1002/jobm.201100335 22359233

[102] Dunlop J, Bowlby M, Peri R, Vasilyev D, Arias R. High-throughput electrophysiology: an emerging paradigm for ion-channel screening and physiology. Nat Rev Drug Discov. 2008;7(4):358–68. doi: 10.1038/nrd2552 18356919

[103] Kryshtafovych A, Schwede T, Topf M, Fidelis K, Moult J. Critical assessment of methods of protein structure prediction (CASP)-Round XIII. Proteins. 2019;87(12):1011–20. doi: 10.1002/prot.25823 31589781 PMC6927249

[104] Agu PC, Afiukwa CA, Orji OU, Ezeh EM, Ofoke IH, Ogbu CO, et al. Molecular docking as a tool for the discovery of molecular targets of nutraceuticals in diseases management. Sci Rep. 2023;13(1):13398. doi: 10.1038/s41598-023-40160-2 37592012 PMC10435576

[105] Sudha R, Katiyar A, Katiyar P, Singh H, Prasad P. Identification of potential drug targets and vaccine candidates in Clostridium botulinum using subtractive genomics approach. Bioinformation. 2019;15(1):18–25. doi: 10.6026/97320630015018 31359994 PMC6651033

[106] Talukdar S, Zutshi S, Prashanth KS, Saikia KK, Kumar P. Identification of potential vaccine candidates against Streptococcus pneumoniae by reverse vaccinology approach. Appl Biochem Biotechnol. 2014;172(6):3026–41. doi: 10.1007/s12010-014-0749-x 24482282 PMC7090528

[107] Mehla K, Ramana J. Novel Drug Targets for Food-Borne Pathogen Campylobacter jejuni: An Integrated Subtractive Genomics and Comparative Metabolic Pathway Study. OMICS. 2015;19(7):393–406. doi: 10.1089/omi.2015.0046 26061459 PMC4505767

[108] Islam MR, Hosen MI, Zaman A, Islam MO. Structural, functional and molecular docking study to characterize GMI1 from Arabidopsis thaliana. Interdiscip Sci. 2013;5(1):13–22. doi: 10.1007/s12539-013-0153-1 23605636

[109] Mora M, Veggi D, Santini L, Pizza M, Rappuoli R. Reverse vaccinology. Drug Discov Today. 2003;8(10):459–64. doi: 10.1016/s1359-6446(03)02689-8 12801798

[110] Shiragannavar SS, Shettar AK, Madagi SB, Sarawad S. Subtractive genomics approach in identifying polysacharide biosynthesis protein as novel drug target against Eubacterium nodatum. Asian J Pharm Pharmacol. 2019;5(2):382–92. doi: 10.31024/ajpp.2019.5.2.24

[111] Kumar A, Thotakura PL, Tiwary BK, Krishna R. Target identification in Fusobacterium nucleatum by subtractive genomics approach and enrichment analysis of host-pathogen protein-protein interactions. BMC Microbiol. 2016;16:84. doi: 10.1186/s12866-016-0700-0 27176600 PMC4866016

[112] Hossain T, Kamruzzaman M, Choudhury TZ, Mahmood HN, Nabi AHMN, Hosen MI. Application of the subtractive genomics and molecular docking analysis for the identification of novel putative drug targets against Salmonella enterica subsp. enterica serovar poona. Biomed Res Int. 2017;2017:3783714. doi: 10.1155/2017/3783714 28904956 PMC5585685

[113] Kumar Jaiswal A, Tiwari S, Jamal SB, Barh D, Azevedo V, Soares SC. An In Silico identification of common putative vaccine candidates against treponema pallidum: a reverse vaccinology and subtractive genomics based approach. Int J Mol Sci. 2017;18(2):402. doi: 10.3390/ijms18020402 28216574 PMC5343936

[114] Hossain M, Chowdhury DUS, Farhana J, Akbar MT, Chakraborty A, Islam S, et al. Identification of potential targets in Staphylococcus aureus N315 using computer aided protein data analysis. Bioinformation. 2013;9(4):187–92. doi: 10.6026/97320630009187 23519164 PMC3602888

[115] Kaur H, Kalia M, Taneja N. Identification of novel non-homologous drug targets against Acinetobacter baumannii using subtractive genomics and comparative metabolic pathway analysis. Microb Pathog. 2021;152:104608. doi: 10.1016/j.micpath.2020.104608 33166618

[116] Al-Ouqaili MTS, Jal’oot AS, Badawy AS. Identification of an oprD and bla(IMP) gene-mediated carbapenem resistance in Acinetobacter baumannii and Pseudomonas aeruginosa among patients with wound infections in Iraq. Asian J Pharm. 2019;12.

[117] Khan MdT, Mahmud A, Iqbal A, Hoque SF, Hasan M. Subtractive genomics approach towards the identification of novel therapeutic targets against human Bartonella bacilliformis. Informatics in Medicine Unlocked. 2020;20:100385. doi: 10.1016/j.imu.2020.100385

[118] Jamal A, Jahan S, Choudhry H, Rather IA, Khan MI. A subtraction genomics-based approach to identify and characterize new drug targets in Bordetella pertussis: Whooping Cough. Vaccines (Basel). 2022;10(11):1915. doi: 10.3390/vaccines10111915 36423010 PMC9692278

[119] D’Souza SE, Khan K, Uddin R. Proteogenomic analysis of Serratia marcescens using computational subtractive genomics approach. PLoS One. 2023;18(4):e0283993. doi: 10.1371/journal.pone.0283993 37036837 PMC10085029

[120] Naorem RS, Pangabam BD, Bora SS, Goswami G, Barooah M, Hazarika DJ, et al. Identification of Putative Vaccine and Drug Targets against the Methicillin-Resistant Staphylococcus aureus by Reverse Vaccinology and Subtractive Genomics Approaches. Molecules. 2022;27(7):2083. doi: 10.3390/molecules27072083 35408485 PMC9000511

[121] Suresh A, Srinivasarao S, Khetmalis YM, Nizalapur S, Sankaranarayanan M, Gowri Chandra Sekhar KV. Inhibitors of pantothenate synthetase of Mycobacterium tuberculosis - a medicinal chemist perspective. RSC Adv. 2020;10(61):37098–115. doi: 10.1039/d0ra07398a 35521286 PMC9057165

[122] Rahman N, Shah M, Muhammad I, Khan H, Imran M. Genome-wide core proteome analysis of brucella melitensis strains for potential drug target prediction. Mini Rev Med Chem. 2021;21(18):2778–87. doi: 10.2174/1389557520666200707133347 32634082

[123] Judson N, Mekalanos JJ. TnAraOut, a transposon-based approach to identify and characterize essential bacterial genes. Nat Biotechnol. 2000;18(7):740–5. doi: 10.1038/77305 10888841

[124] Georrge JJ, Umrania VV. Subtractive genomics approach to identify putative drug targets and identification of drug-like molecules for beta subunit of DNA polymerase III in Streptococcus species. Appl Biochem Biotechnol. 2012;167(5):1377–95. doi: 10.1007/s12010-012-9620-0 22415782

[125] Fatoba AJ, Okpeku M, Adeleke MA. Subtractive Genomics Approach for Identification of Novel Therapeutic Drug Targets in Mycoplasma genitalium. Pathogens. 2021;10(8):921. doi: 10.3390/pathogens10080921 34451385 PMC8402164

[126] Fatoba AJ, Fatoba DO, Babalola SO. Pangenome and subtractive genomic analysis of Clostridioides difficile reveals putative drug targets. J Proteins Proteom. 2022;13(4):247–56. doi: 10.1007/s42485-022-00097-y

[127] Xu G, Tang X, Shang X, Li Y, Wang J, Yue J, et al. Identification of immunogenic outer membrane proteins and evaluation of their protective efficacy against Stenotrophomonas maltophilia. BMC Infect Dis. 2018;18(1):347. doi: 10.1186/s12879-018-3258-7 30053835 PMC6062925

[128] Leow CY, Kazi A, Hisyam Ismail CMK, Chuah C, Lim BH, Leow CH, et al. Reverse vaccinology approach for the identification and characterization of outer membrane proteins of Shigella flexneri as potential cellular- and antibody-dependent vaccine candidates. Clin Exp Vaccine Res. 2020;9(1):15–25. doi: 10.7774/cevr.2020.9.1.15 32095437 PMC7024733

[129] Neu HC, Gootz TD. Antimicrobial chemotherapy. In: Baron S, editor. Medical microbiology. 4th ed. Galveston (TX): University of Texas Medical Branch at Galveston. 1996. 21413283

[130] Li H, Sun X, Cui W, Xu M, Dong J, Ekundayo BE, et al. Computational drug development for membrane protein targets. Nat Biotechnol. 2024;42(2):229–42. doi: 10.1038/s41587-023-01987-2 38361054

[131] Fekkes P, Driessen AJ. Protein targeting to the bacterial cytoplasmic membrane. Microbiol Mol Biol Rev. 1999;63(1):161–73. doi: 10.1128/MMBR.63.1.161-173.1999 10066835 PMC98961

[132] Nielsen DW, Ricker N, Barbieri NL, Allen HK, Nolan LK, Logue CM. Outer membrane protein A (OmpA) of extraintestinal pathogenic Escherichia coli. BMC Res Notes. 2020;13(1):51. doi: 10.1186/s13104-020-4917-5 32005127 PMC6995065

[133] Grund ME, Soo JC, Cote CK, Berisio R, Lukomski S. Thinking Outside the Bug: Targeting Outer Membrane Proteins for Burkholderia Vaccines. Cells. 2021;10(3):495. doi: 10.3390/cells10030495 33668922 PMC7996558

[134] Ansari H, Tahmasebi-Birgani M, Bijanzadeh M, Doosti A, Kargar M. Study of the immunogenicity of outer membrane protein A (ompA) gene from Acinetobacter baumannii as DNA vaccine candidate in vivo. Iran J Basic Med Sci. 2019;22(6):669–75. doi: 10.22038/ijbms.2019.30799.7427 31231495 PMC6570755

[135] Hussein RA, Al-Kubaisy SH, Al-Ouqaili MTS. The influence of efflux pump, outer membrane permeability and β-lactamase production on the resistance profile of multi, extensively and pandrug resistant Klebsiella pneumoniae. J Infect Public Health. 2024;17(11):102544. doi: 10.1016/j.jiph.2024.102544 39321604

[136] Solanki V, Tiwari V. Subtractive proteomics to identify novel drug targets and reverse vaccinology for the development of chimeric vaccine against Acinetobacter baumannii. Sci Rep. 2018;8(1):9044. doi: 10.1038/s41598-018-26689-7 29899345 PMC5997985

[137] Al-Ouqaili MTS, Hussein RA, Majeed YH, Al-Marzooq F. Study of vacuolating cytotoxin A (vacA) genotypes of ulcerogenic and non-ulcerogenic strains of Helicobacter pylori and its association with gastric disease. Saudi J Biol Sci. 2023;30(12):103867. doi: 10.1016/j.sjbs.2023.103867 38020230 PMC10663908

[138] AlQadeeb H, Baltazar M, Cazares A, Poonpanichakul T, Kjos M, French N, et al. The *Streptococcus agalactiae* LytSR two-component regulatory system promotes vaginal colonization and virulence in vivo. Microbiol Spectr. 2024;12(11):e0197024. doi: 10.1128/spectrum.01970-24 39400158 PMC11537067

[139] Mascher T, Helmann JD, Unden G. Stimulus perception in bacterial signal-transducing histidine kinases. Microbiol Mol Biol Rev. 2006;70(4):910–38. doi: 10.1128/MMBR.00020-06 17158704 PMC1698512

[140] Chaffin DO, Beres SB, Yim HH, Rubens CE. The serotype of type Ia and III group B streptococci is determined by the polymerase gene within the polycistronic capsule operon. J Bacteriol. 2000;182(16):4466–77. doi: 10.1128/JB.182.16.4466-4477.2000 10913080 PMC94618

[141] Cieslewicz MJ, Chaffin D, Glusman G, Kasper D, Madan A, Rodrigues S, et al. Structural and genetic diversity of group B streptococcus capsular polysaccharides. Infect Immun. 2005;73(5):3096–103. doi: 10.1128/IAI.73.5.3096-3103.2005 15845517 PMC1087335

[142] Pinzi L, Rastelli G. Molecular Docking: Shifting Paradigms in Drug Discovery. Int J Mol Sci. 2019;20(18):4331. doi: 10.3390/ijms20184331 31487867 PMC6769923

[143] Chowdhury ARK, Billah SM, Meem MB, Mahi MH, Zaman T, Onia SK, et al. In silico Identification and Characterization of Novel Drug Targets in Treponema denticola (strain ATCC 35405 / DSM 14222 / CIP 103919 / JCM 8153 / KCTC 15104): A Subtractive Genomics Approach. Comm Based Med J. 2024;13(2):251–64. doi: 10.3329/cbmj.v13i2.75317

[144] Chowdhury ZM, Jamal TB, Ahammad I, Bhattacharjee A, Lamisa AB, Jani JM, et al. Identification of repurposable drug targets in Mycoplasma pneumoniae using subtractive genomics, molecular docking and dynamics simulation. Heliyon. 2023;9(11):e21466. doi: 10.1016/j.heliyon.2023.e21466 38034688 PMC10682543

